# The Impact of Vitamin D Supplementation on Vitamin D Level, Urinary Calcium Excretion and Bone Density in Patients with Hypercalciuria and Vitamin D Deficiency − Preliminary Report

**DOI:** 10.34763/devperiodmed.20182202.144152

**Published:** 2018-06-30

**Authors:** Joanna Milart, Katarzyna Jobs, Małgorzata Tłustochowicz, Milena Pogonowska, Bolesław Kalicki

**Affiliations:** 1 Department of Paediatrics, Paediatric Nephrology and Allergology, Military Institute of Medicine, Warsaw, Poland; 2 Department of Internal Diseases and Reumatology, Military Institute of Medicine, Warsaw, Poland

**Keywords:** hypercalciuria, urolithiasis, children, vitamin D, bone density, hiperkalciuria, kamica układu moczowego, dzieci, witamina D, gęstość kości

## Abstract

**Aim:**

To study the impact of vitamin D supplementation on vitamin D concentration in plasma, calcium urinary excretion and bone density in patients with urolithiasis in the course of idiopathic hypercalciuria and with a low vitamin D level.

**Materials and method:**

Prospective analysis concerning 28 patients (16 boys, 12 girls) aged 6-14 years (average 10.4) in terms of urinary calcium excretion (mg/kg/day and Ca/Creatinine ratio in morning urine sample), 25OHD blood level after 3, 6, 9 and 12 months of individually recommended doses of vitamin D supplementation (400 IU or 800 IU). The doses were determined on the basis of 25 (OH) D deficiency. The children were on a normocalcemic diet.

The bone mineral density of the patients was assessed before and after 12 months of vitamin D use at the aforementioned doses.

**Results:**

There was no statistically significant correlation between 25 (OH) D plasma concentration and urinary calcium excretion measured on Ca /Creatinine ratio in daily urine collection and Ca/Creatinine ratio in the morning urine sample.

No statistically significant change in calcium excretion was noted (measured by calciuria in daily urine collection and the calcium to creatinine ratio in the morning urine sample). A statistically significant increase in vitamin D plasma concentration was observed. Improvement in bone mineral density was not statistically significant.

**Conclusions:**

Supplementation of vitamin D in the children with idiopathic hypercalciuria and urolithiasis who were examined seems to be safe. The decision to start treatment and the selection of the vitamin D dose should be considered individually.

Patients with urolithiasis should be carefully monitored for calcium/phosphate metabolism parameters and the activity of the disease.

Supplementation of low doses vitamin D in the children examined did not improve bone mineral density.

## Streszczenie

**Cel:**

Próba oceny wpływu podaży witaminy D na jej stężenie w krwi, wydalanie wapnia z moczem oraz na gęstość kości u pacjentów z kamicą w przebiegu hiperkalciurii idiopatycznej oraz z niskimi stężeniami 25(OH)D.

**Materiał i metody:**

Do badania zakwalifikowano 28 dzieci (16 chłopców, 12 dziewczynki) w wieku 6-14 lat (średnio 10,4). Prospektywnie oceniano wydalanie wapnia − kalciurię (straty dobowe, współczynnik wapniowo-kreatyninowy (Ca/Kr)) oraz stężenie 25(OH)D w surowicy po 3, 6, 9 i 12 miesiącach podaży witaminy D w dawce początkowej 400 j.m. lub 800 j.m. na dobę. Dawki były dobierane w oparciu o wielkość niedoboru 25(OH)D. Wszystkie dzieci pozostawały na diecie z normalną zawartością wapnia. Badaniem densytometrycznym oceniono gęstość mineralną kości przed i po 12 miesiącach stosowania powyższych dawek witaminy D.

**Wyniki:**

Nie wykazano istotnych statystycznie korelacji między stężeniem 25(OH)D i wydalaniem wapnia ocenianym wskaźnikiem Ca/Kr z dobowej zbiórki moczu i z drugiej porcji moczu po nocy. U badanych nie stwierdzono także statystycznie istotnego wzrostu kalciurii (wydalanie dobowe i współczynnik Ca/ Kr). Stwierdzono istotny statycznie wzrost stężenia 25(OH)D w surowicy. Wzrost gęstości kości nie był istotny statystycznie.

**Wnioski:**

Stosowanie preparatów witaminy Du badanych przez nas dzieci z hiperkalciurią idiopatyczną i kamicą układu moczowego wydaje się być bezpieczne. Decyzja o włączeniu leczenia powinna być rozpatrywana indywidualnie. Pacjentom należy zalecać dokładne monitorowanie parametrów gospodarki wapniowo-fosforanowej i aktywności kamicy układu moczowego. Suplementacja niskimi dawkami witaminy Du badanych dzieci nie poprawia gęstości kości.

## Introduction

A significant increase in the incidence of urolithiasis among the adult and pediatric population has been observed in recent years. This may be due to environmental factors, such as changing eating habits, which promote obesity and an inadequate daily intake of water.

Metabolic disorders are found in many children with urolithiasis and may promote the onset of various diseases. The most common one is idiopathic hypercalciuria (IH). It is estimated that it coexists with kidney stones in 30% - 60% of the adults and 40-80% of the children affected [1, 2, 3, 4, 5, 6, 7]. Idiopathic hypercalciuria is characterized by normocalcaemia and hypercalciuria and an absence of circumstances that cause increased calcium excretion (i.e. steroid therapy, use of drugs increasing calcium excretion or hyperparathyroidism). Hypercalciuria is usually defined as daily calcium excretion above 4 mg per kilogram of body weight.

Random Ca/Creatinine ratio is a useful tool for diagnosing IH. It is a quotient of calcium and creatinine excretion in the second morning urine sample. The reference values for this ratio vary with age, latitude and range from 0.8 in infants to 0.2 in adults. Genetic predisposition to IH is suspected. Studies have shown an increased incidence of nephrolithiasis and hypercalciuria among the first-degree relatives of patients with kidney stones [1, 7, 8]. In addition, diet has a large effect on hypercalciuria in individuals with IH. Reduced intake of calcium and magnesium in the diet leads to an increase in free oxalates in the intestines, thus increased oxalate absorption and increased urinary excretion, which induce the formation of stones in the urinary tract [1, 9, 10]. Therefore, a normocalcaemic regimen should be recommended to IH patients [9, 11, 12].

Vitamin D deficiency is commonly observed in the Polish population (summer months included). This may be due to changes of lifestyle and work habits (leading to lower sun exposure) and a wide use of protection against ultraviolet radiation (sunscreen). Improved methods of assessment of vitamin D supplies and the relatively low price of the test have made it possible to monitor and correct vitamin D deficiencies. Vitamin D supplementation is recommended especially in autumn and winter months. Prophylactic doses have been established for the population of our latitude [11, 13].

However, vitamin D supplementation may be harmful in some patient groups. Until recently treatment was considered not safe in disorders of calcium and phosphate metabolism (urolithiasis and hypercalciuria among them). Vitamin D deficiency often coexists with these disorders. The serum level of liver hydroxylated 25(OH)D is an indication of the body’s vitamin reserves. Idiopathic hypercalciuria was proven to coexist with bone mineralization disorders (osteopenia and osteoporosis – low bone mass and impaired bone structure), hence a greater risk of bone fracture in the pediatric population [11, 14, 15, 16]. Thus, the question arises whether vitamin D administration does not increase calciuria and hence promote the formation of new stones in the urinary tract among patients with IH induced urolithiasis. Another important issue is the effect of administering strictly fixed vitamin D doses on bone density in this group of patients [10, 11, 14].

## The aim

The aim of the study was to evaluate the impact of vitamin D supplementation on vitamin D concentration in plasma, calcium urinary excretion and bone density in patients with urolithiasis in the course of idiopathic hypercalciuria and with a low vitamin D level.

## Materials and method

This study included regular patients of the Pediatric, Nephrology and Allergology Department at the Military Institute of Medicine with idiopathic hypercalciuria induced urolithiasis, in which serum vitamin D concentration corresponded to an average deficiency (10-20 ng/ ml) or suboptimal concentration (20-30 ng/ml) [11]. Excluded from the study were children with chronic kidney disease, urinary tract infections, urinary tract defects, systemic diseases, bone diseases (other than osteopenia), endocrine disorders and those using glucocorticosteroids.

It was planned that if control studies revealed a significant increase in calciuria or the formation of new deposits, then the patient would return to the previous treatment regimen (the vitamin D supplement would be withdrawn). One of the patients was treated with hydrochlorothiazide, which was discontinued at the beginning of the study. Other patients with hypercalciuria did not receive any additional treatment before and at the time of the study.

Prospective analysis included 28 patients (16 boys, 12 girls) aged 6-14 years (average 10.4). Analysis was performed of calciuria (daily calcium excretion, random Ca/Creatinine ratio), and the level of 25OHD after 3, 6, 9 and 12 months of vitamin D supplementation. Doses were determined on the basis of 25(OH)D deficiency: 400 IU for patients with 25(OH)D 20-30ng/ml; 800 IU for those with 25(OH)D 10-20 ng/ml.

Urinary calcium excretion was measured by photometric absorbance. The Ca/Creatinine ratio was calculated (quotient of calcium and creatinine excretion in the second morning urine sample).

Total concentration of 25-hydroxyvitamin D was measured with the Dia-Sorin LIAISON® analyzer, which uses chemiluminescent immunoassays (CLIA) to determine the level of 25OHD in serum and plasma.

Statistical significance was measured only between the initial and 12-month visit.

Bone mineral density of patients was assessed before and after 12 months of vitamin D use at the aforementioned doses. Bone densitometry was performed with HOLOGIC model Delphi W (S/N 70608) in the Whole Body projection. Bone mineral density (BMD) in g/cm^2^ was then used to calculate the T-score using the formula: Z-score = (BMD patient-BMD adjusted for age)/SD. Osteopenia is defined as Z-score between (-2) and (-1), osteoporosis is defined as Z score> (-2) [18].

Calcium/phosphate metabolism parameters were initially evaluated: serum creatinine (Cr), calcium (Ca), phosphorus (P), magnesium (Mg), sodium (Na), uric acid (Ua), alkaline phosphatase (ALP), parathyroid hormone (PTH), glomerular filtration rate (eGFR); excretion of creatinine (Cr), calcium (Ca), magnesium (Mg), phosphorus (P) and uric acid (Ua) were evaluated in the 24h-urine collection and second morning urine sample. Urinalysis and urine culture was also performed. The correct test results were used to classify patients into the study group (results not included here).

In the first period of observation, before the decision about vitamin D supplementation we evaluated the:

− serum level of liver hydroxylated 25(OH)D,
− calcium excretion in the 24h urine collection and second morning urine sample,
− full body densitometry. Results were given as mean and standard deviation (Z-score).

Then the patients received vitamin D at a dose of 400 IU or 800 IU daily, depending on initial 25(OH) D concentration. Children with moderate vitamin D deficiency (10-20ng/ml) received 800 IU daily, children with suboptimal vitamin D concentrations (20-30 ng/ ml) received 400 IU daily.

Every 3 months, during each follow-up visit, evaluation was conducted of:

− serum 25 (OH) D concentration,
− calcium excretion in 24h urine collection, and Ca/ Creatinine ratio in second morning urine sample.

During observation, vitamin D doses were adjusted to the seasons and concentrations of 25 (OH) D in each patient, using only a dose of 400 IU or 800 IU. Calcium excretion was not taken into consideration in the criteria of changing doses.

After 12 months of vitamin D intake, full body bone densitometry was performed alongside the blood and urine tests.

Additionally, all the patients underwent a renal ultrasound every 3 months to assess new deposit formation. The disease activity is still studied and will be presented in the future.

The results were statistically analyzed based on the software StatSoft, Inc. (2014) STATISTICA (data analysis software system), version 12. Before the analysis began, the data were initially verified using the normality diagram of the distribution, and finally using the normality test of Kolmogorow-Smirnov and Liliefors. The distribution of data for analysis was normal, so Pearson’s linear correlation coefficient was used. Statistically significant values were those for which p <0.05.

## Results

No patient in the study group required withdrawal of vitamin D supplementation.

**Fig. 1:**
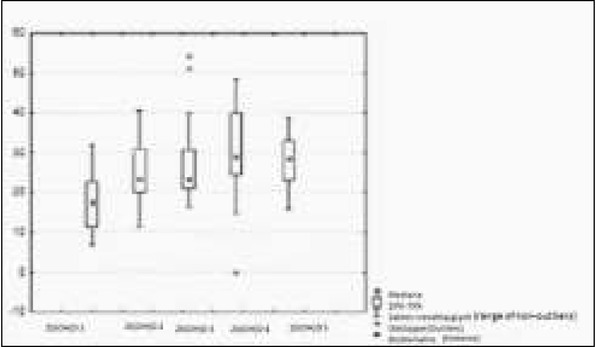
Concentration of 25 (OH) D at the beginning and after 3, 6, 9 and 12 months of observation. Ryc. 1. Stężenie 25(OH)D wyjściowo i po 3, 6, 9 i 12 miesiącach obserwacji.

**Fig. 2:**
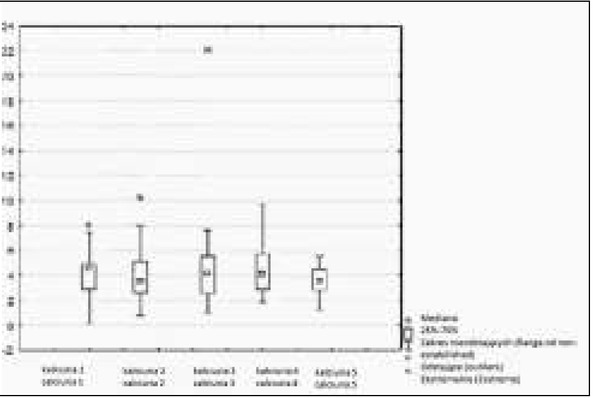
Urinary excretion of calcium in daily urine collection before treatment and after 3, 6, 9 and 12 months of observation. Ryc. 2. Wydalanie wapnia z moczem w DZM wyjściowo i po 3, 6, 9 i 12 miesiącach obserwacji.

**Fig. 3:**
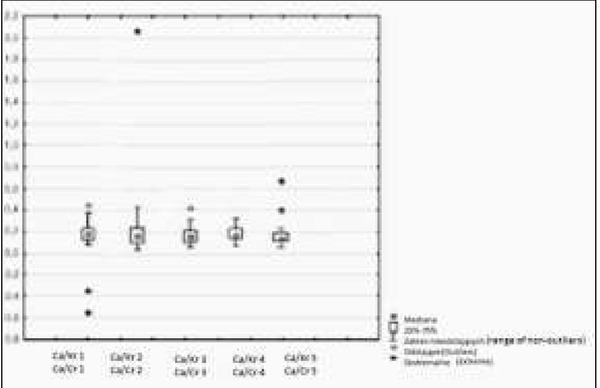
Ca/Creatinine ratio in the second morning urine sample before treatment and after 3, 6, 9 and 12 months of observation. Ryc. 3. Wartości współczynnika Ca/Kr z 3 godzinnej ZM wyjściowo i po 3, 6, 9 i 12 miesiącach obserwacji.

The results are shown in Figures 1, 2 and 3 and in Table I.

**Table I:** Mean values and range of calciuria, Ca/Creatinine ratio, 25OHD serum concentration and densitometry results according to the length of treatment. Tabela I. Wartości średnie i zakres kalciurii, współczynnika Ca/Kr, stężenia 25OHD oraz wyniki densytometrii w zależności od czasu leczenia.

	Before treatment *Przed leczeniem*		After 3 months *Po 3 miesiącach*		After 6 months *Po 6 miesiącach*		After 9 months *Po 9 miesiącach*		After 12 months *Po 12 miesiącach*		Statistical significance before teratment and after 12 months (p<0.05) *Znamienność statystyczna przed leczeniem i po 12 miesiącach (p<0,05)*
	*Av*. *Śr*.	Range zakres	Av. Śr.	Range zakres	Av. Śr.	Range zakres	Av. Śr.	Range zakres	Av. Śr.	Range zakres	
Calciuria (mg/kg/daily) *Kalciuria (mg/kg/24h)*	4.15	0.17-7.44	3.96	0.79-10.2	3.92	0.93-7.6	4.65	1.83 -7.78	3.57	1.24-5.54	0.61
Ca/Creatinine ratio *Wskaźnik* *Ca/Kr*	0.22	0.08- 0.44	0.17	0.04-0.26	0.16	0.71-0.29	0.18	0.06-0.29	0.18	0.06-0.24	0.05
25(OH)D (ng/ml)	17.8	6.8-31.8	24.7	11.0- 40.2	26.6	16.1-54.2	31.14	14.6-48.2	27.59	15.6-38.5	0.00005
Densitometry (Z-score) *Densytometria*	(-0.730)	(-1.98)–(1.46)							(-0.850)	(-1.870)–(2.01)	0.68

Ca/Creatinine – calcium to creatinine ratio; 25(OH)D – 25 hydroxyvitamin D, av – average Ca/Kr – wskaźnik wapniowo–kreatyninowy; 25(OH)D – 25 hydroksywitamina D, śr - średnia

Urine calcium excretion, evaluated in mg/kg/day, initially amounted to 4.15 (0.17-7.44); after 3 months, the excretion decreased to an average of 3.96 (0.79 to 10.2); after 6 months it did not change significantly by an average of 3.92 (from 0.93 to 7.6); after 9 months it rose to an average of 4.65 (from 1.83 to 7.78); whereas after 12 months it decreased to an average of 3.57 (from 1.24 to 5.54). There was no statistically significant increase in urinary calcium excretion measured before treatment and after 12 months of treatment (p=0,61).

Calcium excretion measured by Ca/ Creatinine ratio was initially 0.22 (0.083 to 0.44); after 3 months of vitamin D supplementation Ca/Creatinine ratio decreased to an average of 0.17 (from 0.041 to 0.258); after 6 months its value did not increase and was on average 0.16 (from 0.71 to 0.29); after 9 months an average of 0.18 was observed (from 0.063 to 0.298); after 12 months − an average 0.18 (from 0.06 to 0.24) was registered. Urinary calcium excretion measured with Ca/Creatinine at the beginning of the study and after 12 months of treatment did not change in a statistically signiftcant way, it seemed to be even lower than initially (p=0.05).

Initially 25OHD concentration (in ng/ml) in the patients was on average 17.8 (6.8 to 31.8); after 3 months of supplementation it increased to an average of 24.7 (from 11 to 40.2); after 6 months it was on average 26.2 (16.1 to 54.2); after 9 months the average was 31.14 (from 14.6 to 48.2); after 12 months it averaged 27.59 (from 15.6 to 38.5). The increase in 25 (OH) D after 12 months of treatment was statistically significant (p=0,00005) (Figure 1, Table I).

**Fig. 4:**
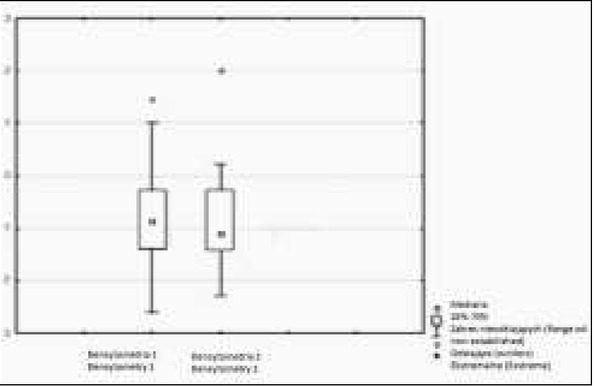
Z-score in densitometry before treatment and after 12 months of observation. Ryc. 4. Wartość współczynnika Z-score w densytometrii wyjściowo i po 12 miesiącach obserwacji.

For the evaluation of densitometry, the Whole Body Z score was used, which before treatment was on average (-1.79) [from (-1.98) to (1.46)], whereas after one year of observation, an average of (-0.85) [from (-1.87) to (2.01)] was observed. A change in the Whole Body Z-score after 12 months of vitamin D supplementation was not statistically significant (p=0.68). There was no statistically significant correlation between vitamin D 25 (OH) concentration and calciuria in mg per kg body weight in daily urine collection (Figure 1, Figure 2, Table II). No statistically significant correlation was found between 25 (OH) D concentration and Ca/Creatinine ratio in the daily and second morning urine sample (Figure 1, Figure 3, Table III). The increase in bone density was not statistically significant (Table I, IV).

**Table II:** The correlation between vitamin D and calciuria before treatment and after 3, 6, 9 and 12 months of observation. Tabela II. Zależność między stężeniem witaminy D a kalciurią wyjściowo i po 3, 6, 9 i 12 miesiącach obserwacji.

	**Correlationslndicated correlation coefficients are significant with p<0.05** ***Korelacje*** ***Oznaczone wsp. korelacji są istotne z p<0,05***				
	Calciuria before treatment *kalciuria wstępnie*	Calciuria after 3 months *kalciuria po 3 m-cach*	Calciuria after 6 months *kalciuria po 6 m- cach*	Calciuria after 9 months *kalciuria po 9 m-cach*	Calciuri aafter 12 months *kalciuria po 12 m-cach*
25(OH)D Before treatment *25(OH)D wstępnie*	r=0.28 p=0.29				
25(OH)D after 3 months *25(OH)D po 3 miesiącach*		r=-0.06 p=0.78			
25(OH)D after 6 months *25(OH)D po 6 miesiącach*			r=-0.23 p=0.22		
25(OH)D after 9 months *25(OH)D po 9 miesiącach*				r=0.27 p=0.20	
25(OH)D after 12 months *25(OH)D po 12 miesiącach*					r=-0.32 p=0.22

r– correlation, p – statistical significance, 25(OH)D 25hydroxyvitamin D r – korelacja, p – istotność statystyczna, 25(OH)D 25hydroksywitamina D

**Table III:** The relationship between vitamin D concentration and Ca/Creatinine ratio in daily urine collection before treatment and after 3, 6, 9 and 12 months of observation. Tabela III. Zależność między stężeniem witaminy D a współczynnikiem Ca/Kr z DZM wyjściowo i po 3, 6, 9 i 12 miesiącach obserwacji.

	Correlations Indicated correlation coefficients are significant with p<0.05 *Korelacje* *Oznaczone wsp. korelacji są istotne z p<0,05*				
	Ca/Creatinine before treatment *Ca/Kr wstępnie*	Ca/Creatinine after 3 months *Ca/Kr po 3 m-cach*	Ca/Creatine after 6 months *Ca/Kr po 6 m-cach*	Ca/Creatinie after 9 months *Ca/Kr po 9 m-cach*	Ca/Creatinineafafter 12 months *Ca/Kr po 12 m-cach*
25(OH)D before treatment *25(OH)D wstępnie*	r=0.19 p=0.33				
25(OH)D after 3 months *25(OH)D po 3 miesiącach*		r=-0.19 p=0.33			
25(OH)D after 6 months *25(OH)D po 6 miesiącach*			r=0.22 p=0.90		
25(OH)D after 9 months *25(OH)D po 9 miesiącach*				r=-0.31 p=0.16	
25(OH)D after 12 months *25(OH)D po 12 miesiącach*					r=0.15 p=0.57

r –correlation, p – statistical significance, 25(OH)D 25hydroxyvitamin D, Ca/Creatynine – calcium to creatinine ratio r– korelacja, p– istotność statystyczna, 25(OH)D 25hydroksywitamina D, Ca/Kr – współczynnik wapniowo-kreatyninowy

**Table IV:** The relationship between 25 (OH) D serum concentration and Z score in densitometry before treatment and after 12 months of follow-up. Tabela IV. Zależność między stężeniem 25(OH)D a współczynnikiem Z score w densytometrii wyjściowo i po 12 miesiącach obserwacji.

	Correlations Indicated correlation coefficients are significant with p<0.05 *Korelacje Oznaczone wsp. korelacji są istotne z p<0,05*	
	Densitometry 1 *Densytometria 1*	Densitometry 2 *Densytometria 2*
25(OH)D Before supplementation of vitamin D *25(OH)D przed włączeniem wit. D*	r=0.15 p=0.45	
25(OH)D After 12 months of vitamin D supplementation *25(OH)D po 12 miesiącach leczenia*		r=0.23 p=0.39

r − correlation, p − statistical significance, 25(OH)D − 25hydroxyvitamin D, densitometry 1 − before treatment, densitometry 2 − after 12 month of vitamin D supplementatio r − korelacja, p − istotność statystyczna, 25(OH)D − 25hydroksywitamina D, densytometria 1 − przed leczeniem, densytometria 2 − po 12 miesiącach suplementacji witaminy D

## Discussion

Until recently, the administration of vitamin D to patients with urinary stones was considered harmful and assumed to lead to an increase in the disease’s activity [20]. Expanded interest in the value of vitamin D and its pleiotropic activity led to research into the advantageous effects of vitamin D, especially in patients with urolithiasis coexisting with decreased bone density. In accordance with generally accepted recommendations, IH patients should have a normocalcaemic regimen [9, 11, 12].

The effect of the administration of vitamin D on the activity of urolithiasis is still controversial. There are no unequivocal studies about the influence of vitamin D supplementation on urolithiasis severity. There is also no clear opinion about the effect of vitamin D supplementation on bone density in these patients.

In the literature there are only few, relatively recent studies referring to adults, and these publications present contradictory results [10, 11, 14].

The Department of Pediatrics, Pediatric Nephrology and Allergology at the Military Institute of Medicine takes care of the constantly increasing group of children and adolescents with urinary stones in whom serum 25 (OH) D concentration is routinely evaluated.

Observation of the common vitamin D deficiency in this group has become the basis for planning the current study with low doses of vitamin D in children with urolithiasis caused by idiopathic hypercalciuria. Vitamin D was administered under the supervision of their calcium metabolism and 25 (OH) D concentration.

This study demonstrated that 12 months of low-dose vitamin D supplementation (400 or 800 IU/day) in children with IH did not significantly affect calciuria as measured by daily calcium excretion and Ca/Creatinine ratio. However, a statistically significant increase in vitamin D serum hepatic metabolite concentration was proven. Low doses of vitamin D did not improve bone density (Z-score) (p=0.68).

Leaf et al. [17] who carried out research on adult populations in the USA did not prove an increase in calcium excretion in patients with urolithiasis through the supplementation of high doses of ergocalciferol (50 IU per week). Johri et al. [18] found that the supplementation of vitamin D increased the excretion of calcium. However, many studies of healthy subjects treated with vitamin D supplementation did not reveal any increased risk for calciuria [19].

Zerwekh is of the an opinion that the effect of vitamin D on the kidneys requires further research, as increased reabsorption of calcium in the renal tubules associated with increased serum vitamin D concentration may affect decreased calcium urinary excretion [20]. Thus, perhaps, there is no physiological evidence for concern that vitamin D administration increases calciuria. All of these references emphasize that even low-dose supplementation of vitamin D results in a significant increase in serum 25 (OH) D concentration [17, 18, 19]. The relationship between urolithiasis and IH and decreased bone density was confirmed. However, there is no clear opinion about the effect of vitamin D supplementation on bone density in pediatric patients. Tasca et al. believe idiopathic hypercalciuria coexists with decreased bone density and it has a strong connection with intestinal absorption, bone metabolism and calcium excretion [8]. Artemiuk et al. conducted a study on a pediatric population with urolithiasis showing a correlation between low vitamin D levels and decreased bone density in the lumbar spine [22].

A limitation of our study is the small population. Pre-qualified patients often did not report for screening, which unfortunately limited the number of those who completed the program.

There is also insufficient insight into vitamin D supplementation regularity, because the preparations were used by patients themselves in home conditions and only the observation of increased 25(OH)D concentration was evidence of accomplishing the recommendations.

It is possible that no improvement in bone density parameters in the presented study is associated with the low vitamin D doses or too short a follow-up period.

In subsequent studies, the use of larger doses of vitamin D should be considered. The influence of these doses on the bone density and urolithiasis activity in children with idiopathic hypercalciuria and osteopenia should be analyzed.

## Conclusions

1. The supplementation of vitamin D in the children with idiopathic hypercalciuria and urolithiasis who were examined seems to be safe.
2. The decision to start treatment and the selection of the vitamin D dose should be considered individually.
3. Patients with urolithiasis should be carefully monitored for calcium/ phosphate metabolism parameters and the activity of the disease.
4. Supplementation of low doses vitamin D in the children examined did not improve bone density.
